# Detection efficacy of [^89^Zr]Zr-PSMA-617 PET/CT in [^68^Ga]Ga-PSMA-11 PET/CT-negative biochemical recurrence of prostate cancer

**DOI:** 10.1007/s00259-023-06241-0

**Published:** 2023-05-06

**Authors:** Florian Rosar, Fadi Khreish, Robert J. Marlowe, Andrea Schaefer-Schuler, Caroline Burgard, Stephan Maus, Sven Petto, Mark Bartholomä, Samer Ezziddin

**Affiliations:** 1 Department of Nuclear Medicine, Saarland University – Medical Center, Kirrberger Str. 100, Geb. 50, 66421 Homburg, Germany; 2 Spencer-Fontayne Corporation, Jersey City, NJ USA

**Keywords:** Prostate cancer, Biochemical recurrence, Positron emission tomography/computed tomography (PET/CT), Prostate-specific membrane antigen (PSMA), Zirconium-89 (^89^Zr)

## Abstract

**Rationale:**

In patients with biochemical recurrence of prostate cancer (BCR), preliminary data suggest that prostate-specific membrane antigen (PSMA) ligand radiotracers labeled with zirconium-89 (^89^Zr; half-life ~ 78.41 h), which allow imaging ≥ 24 h post-injection, detect suspicious lesions that are missed when using tracers incorporating short-lived radionuclides.

**Materials and methods:**

To confirm [^89^Zr]Zr-PSMA-617 positron emission tomography/computed tomography (PET/CT) detection efficacy regarding such lesions, and compare quality of 1-h, 24-h, and 48-h [^89^Zr]Zr-PSMA-617 scans, we retrospectively analyzed visual findings and PET variables reflecting lesional [^89^Zr]Zr-PSMA-617 uptake and lesion-to-background ratio. The cohort comprised 23 men with BCR post-prostatectomy, median (minimum–maximum) prostate-specific antigen (PSA) 0.54 (0.11–2.50) ng/mL, and negative [^68^Ga]Ga-PSMA-11 scans 40 ± 28 d earlier. Primary endpoints were percentages of patients with, and classifications of, suspicious lesions.

**Results:**

Altogether, 18/23 patients (78%) had 36 suspicious lesions (minimum–maximum per patient: 1–4) on both 24-h and 48-h scans (n = 33 lesions) or only 48-h scans (n = 3 lesions). Only one lesion appeared on a 1-h scan. Lesions putatively represented local recurrence in 11 cases, and nodal or bone metastasis in 21 or 4 cases, respectively; 1/1 lesion was histologically confirmed as a nodal metastasis. In all 15 patients given radiotherapy based on [^89^Zr]Zr-PSMA-617 PET/CT, PSA values decreased after this treatment. Comparison of PET variables in 24-h vs 48-h scans suggested no clear superiority of either regarding radiotracer uptake, but improved lesion-to-background ratio at 48 h.

**Conclusions:**

In men with BCR and low PSA, [^89^Zr]Zr-PSMA-617 PET/CT seems effective in finding prostate malignancy not seen on [^68^Ga]Ga-PSMA-11 PET/CT. The higher detection rates and lesion-to-background ratios of 48-h scans versus 24-h scans suggest that imaging at the later time may be preferable. Prospective study of [^89^Zr]Zr-PSMA-617 PET/CT is warranted.

## Introduction

The diagnostic performance of imaging in patients with biochemical recurrence of prostate cancer (BCR) has been substantially improved by positron emission tomography/computed tomography (PET/CT) using radiotracers targeted at prostate-specific membrane antigen (PSMA) [1, 2], a transmembrane glycoprotein overexpressed on the surface of prostate carcincoma cells [3–5]. To date, PSMA-targeted radiotracers that are used in everyday practice comprise PSMA ligands labeled with either of two short-lived radionucludes, gallium-68 (^68^Ga; half-life ~ 1.1 h) or fluorine-18 (^18^F; half-life ~ 1.8 h) [6–12]. Such tracers combine generally good sensitivity and specificity with relatively low radiation exposure and the convenience and workflow efficiency of early image acquisition.

However, PET/CT with ^68^Ga or ^18^F tracers cannot detect suspicious lesions in an appreciable proportion of cases of BCR, especially when prostate-specific antigen (PSA) values are low [13, 14]. For example, our group reported a 74.8% rate of negative [^68^Ga]Ga-PSMA-11 PET/CT in men with PSA levels ≤ 0.2 ng/mL (N = 115) [15], while Afshar-Oromieh et al. reported a 42.5% rate of negative scans in those with PSA > 0.2–0.5 ng/mL (n = 630) and a 27.8% rate in those with PSA > 0.5– ≤ 1.0 ng/mL (n = 526) [2].

To some extent, this diagnostic dilemma may be mitigated when image acquisition using ^68^Ga or ^18^F tracers is performed at additional, delayed time points, e.g., 3 h post-[^68^Ga]Ga-PSMA-11 administration [16]. Nonetheless, certain cases of missed lesions may relate to a key limitation of the short-lived tracers: inability to provide interpretable images beyond several hours post-injection. PET/CT with such tracers thus might not visualize lesions requiring longer times to internalize PSMA-targeted radiopharmaceuticals, e.g., lesions with low PSMA expression or low perfusion [17]. Alternatively, residual urinary tract activity associated with hours-long renal clearance of a short-lived radiopharmaceutical may obscure lesions in sites such as the ureter or urinary bladder [17, 18].

Interest therefore has increased in imaging patients with BCR with tracers that combine PSMA ligands with the radionuclide zirconium-89 (^89^Zr), with its much longer half-life, ~ 78.41 h [13, 17–19]. Building on earlier work by others [20, 21], our group and our collaborators from Radboud University Medical Center/the University of Nijmegen stably conjugated ^89^Zr to PSMA-617 [17]; we then reported on this radiotracer’s biodistribution, organ and whole-body dosimetry, and use in a small number of men with BCR (*N* = 8). We observed that [^89^Zr]Zr-PSMA-617 PET/CT at ≥ 24 h post-injection frequently revealed lesions suspicious for prostate cancer that had been missed on ~ 1-h [^68^Ga]Ga-PSMA-11 PET/CT images [17, 19, 22].

This favorable preliminary clinical experience, and experience reported with additional ^89^Zr-conjugated PSMA-targeted tracers [13, 17, 18], led us to perform the present retrospective analysis of our use of [^89^Zr]Zr-PSMA-617 PET/CT in a larger group of patients with BCR and recent prior negative [^68^Ga]Ga-PSMA-11 PET/CT. Our goal was to confirm the detection efficacy of [^89^Zr]Zr-PSMA-617 PET/CT, and to gather additional data regarding appropriate timing of image acquisition when using this novel modality.

## Materials and methods

### Endpoints

The primary endpoints of this analysis were the percentage of the study sample with clear visual evidence of lesions suspicious for prostate cancer, and the classification of those lesions (local recurrence, lymph node metastasis, bone metastasis) on scans performed 1 h, 24 h, or 48 h after [^89^Zr]Zr-PSMA-617 administration. The 1-h [^89^Zr]Zr-PSMA-617 scans were performed to evaluate early imaging using an ^89^Zr-containing radiotracer, and to allow direct comparison with the conventional [^68^Ga]Ga-PSMA-11 scan; the 24-h and 48-h [^89^Zr]Zr-PSMA-617 scans were performed to assess image acquisition at times that presumably could be used effectively with the long-lived ^89^Zr, but not the short-lived ^68^Ga.

Secondary endpoints were the values on each scan for each suspicious lesion of four key [^89^Zr]Zr-PSMA-617 PET variables reflecting lesional radiotracer uptake or lesion-to-background ratio, and comparison of these variables between scans at different time points. The variables reflecting lesional radiotracer uptake were the maximum and peak standardized uptake values (SUV_max_ and SUV_peak_, respectively) of the lesions; the variables reflecting lesion-to-background ratio were the tumor-to-background ratios of SUV_max_ or SUV_peak_ (TBR_max_ or TBR_peak_, respectively), i.e., the SUV_max_ or SUV_peak_ of the lesion/mean standardized uptake value (SUV_mean_) of the tissue used as background, healthy gluteal muscle.

An additional secondary endpoint of the analysis was short-term safety, i.e., side effects or vital signs abnormalities that we believed to be associated with [^89^Zr]Zr-PSMA-617 PET/CT. Also, we compiled data regarding prostate cancer-related interventions post-[^89^Zr]Zr-PSMA-617 PET/CT and these interventions' results.

### Patients and ethics

The cohort comprised 23 consecutive men with BCR, defined as increasing PSA following radical prostatectomy. These patients underwent imaging for that indication at our center between 27 April 2021 and 22 August 2022. They had negative [^68^Ga]Ga-PSMA-11 PET/CT scans, defined by absence of visual evidence of non-physiological radiotracer uptake. The [^68^Ga]Ga-PSMA-11 PET/CT was performed per standard procedures [23], with images acquired ~ 1 h after administration of a mean ± standard deviation (SD) 148 ± 21 MBq activity of [^68^Ga]Ga-PSMA-11. [^89^Zr]Zr-PSMA-617 PET/CT took place 40 ± 28 (median [minimum–maximum] 35 [6–104]) d thereafter. To avoid a factor that potentially could confound scan interpretation, patients were eligible for this analysis only if they received no treatment for prostate cancer during the interval between [^68^Ga]Ga-PSMA-11 PET/CT and [^89^Zr]Zr-PSMA-617 PET/CT.

Table 1 summarizes study sample characteristics. The patients were generally middle-aged to elderly, with Gleason stage ≤7 disease in about two-thirds. The PSA value (median [minimum–maximum]) was 0.53 [0.12–2.49] ng/mL at [^68^Ga]Ga-PSMA-11 PET/CT and 0.54 [0.11–2.50] ng/mL at [^89^Zr]Zr-PSMA-617 PET/CT. No patient had a history of any malignancy other than prostate cancer.

**Table 1:** Patient characteristics

Characteristic	Value
Age	
Median (min.–max.)	67 (53–77)
PSA [ng/mL], median (min.–max.)	
At [^68^Ga]Ga-PSMA-11 PET/CT	0.53 (0.12–2.49)
At [^89^Zr]Zr-PSMA-617 PET/CT	0.54 (0.11–2.50)
PSA doubling time, % (n)	
< 3 mo	17% (4)
3–6 mo	39% (9)
7–12 mo	22% (5)
> 12 mo	22% (5)
Gleason Score, % (n)	
6	4% (1)
7a	17% (4)
7b	39% (9)
8	22% (5)
9	17% (4)
Primary treatment, % (n)	
Prostatectomy	100% (23)
Additional treatments before PSMA PET/CT, % (n)	
Radiation therapy	35% (8)
ADT	22% (5)
Lymphadenectomy	4% (1)

ADT, androgen deprivation therapy; max., maximum; min., minimum; PSA, prostate-specific antigen, PSMA, prostate-specific membrane antigen

Patients underwent [^89^Zr]Zr-PSMA-617 PET/CT on a compassionate use basis under the German Pharmaceutical Act §13 (2b). Their treating nuclear medicine physicians had direct responsibility for the procedure, including requisitioning the radiopharmaceutical. The analysis was conducted according to the Declaration of Helsinki and was approved by the Institutional Review Board of the Ärztekammer des Saarlandes/Saarbrücken (approval number: 170/22, approval date: 13 September 2022). All patients provided written consent for [^89^Zr]Zr-PSMA-617 PET/CT after receiving comprehensive information regarding the risks of radiation exposure from the procedure, and regarding the potential for side effects of the novel PET agent. The latter information included a summary of adverse events associated to date with current PSMA-targeted radiotracers, and those associated with PSMA radioligand therapy (RLT). The consent also covered use of the resulting data, in de-identified form, for scientific publications. Data regarding 5/23 cases (22%) were reported previously [17, 19, 22].

### [^89^Zr]Zr-PSMA-617 PET/CT

Whole-body images, extending from vertex to mid-femur, were acquired 1 h, 24 h, and 48 h after intravenous injection of a mean ± SD 116 ± 20 (median [minimum–maximum]: 119 [84–163]) MBq of radiotracer, immediately followed by a 500-mL NaCl 0.9% infusion. Patients were instructed to void before each image acquisition. [^89^Zr]Zr-PSMA-617 was made in-house as described previously [19].

All imaging was performed in 3D mode on a Biograph mCT 40 scanner (Siemens Medical Solutions, Knoxville, TN, USA). PET acquisition time was 3 min/bed position for the 1-h scan, 4 min/bed position for the 24-h scan, and 5 min/bed position for the 48-h scan, with an extended field-of-view of 21.4 cm.

For attenuation correction and anatomical localization, low-dose CT was performed employing a 120-keV x-ray tube voltage and tube current modulation using CARE Dose4D software (Siemens Healthineers, Erlangen, Germany), with 30 mAs as the reference. Data were reconstructed with a soft tissue kernel (B31f/Be32) to a slice thickness of 5 mm (increment: 2–4 mm).

Along with attenuation correction, PET emission data underwent decay correction, random correction, and scatter correction. PET images were reconstructed applying an iterative 3-dimensional ordered-subset expectation maximization algorithm (3 iterations; 24 subsets) with Gaussian filtering to a transaxial resolution of 5 mm at full width at half maximum. The matrix size was 200 × 200 and the pixel size, 3.0 mm.

### Interpretation of [^89^Zr]Zr-PSMA-617 PET/CT images and calculation of PET variables

[^89^Zr]Zr-PSMA-617 scans were visually interpreted by consensus by three nuclear medicine physicians (SE, FK, FR) with extensive experience in reading PET/CT images acquired with PSMA-targeted radiotracers. Since image interpretation took place within everyday practice rather than within a clinical trial, readers were not blinded to the patient’s prostate cancer-related and other history. In interpreting the [^89^Zr]Zr-PSMA-617 PET/CT scans, [^68^Ga]Ga-PSMA-11 PET/CT findings were taken into account, as were findings of earlier [^89^Zr]Zr-PSMA-617 scans in the cases of the 24-h and 48-h scans.

Each lesion that appeared to be suspicious for prostate cancer was analyzed using SyngoVia software (Enterprise VB 60, Siemens, Erlangen, Germany) to measure SUV_max_ and SUV_peak_, and calculate TBR_max_ and TBR_peak._ The latter two variables were respectively defined as SUV_max_ or SUV_peak_ of the lesion divided by SUV_mean_ of healthy gluteal muscle, the tissue used as background. SUV_mean_ was calculated within a volume of interest applying a threshold of 20% of SUV_max_.

### Monitoring for potential adverse events related to [^89^Zr]Zr-PSMA-617 PET/CT

We recorded adverse events and clinically-relevant abnormalities in vital signs that we believed to be related to [^89^Zr]Zr-PSMA-617 PET/CT and that were observed by health care professionals, reported by the patient, or both during imaging and up to 4 weeks thereafter. Questions about specific potential side effects as well as open-ended queries about the occurrence of side effects in general were posed to patients in telephone calls made shortly after scanning and/or after the first follow-up visit.

### Statistics

Data are presented as descriptive statistics, including mean ± SD, median (minimum–maximum), and number (percentage) or vice versa, as appropriate. The Wilcoxon matched-pairs signed rank test was used for intra-individual comparison of values for PET variables between scans acquired at different time points.

Prism version 8 (GraphPad Software, San Diego, USA) was used for the statistical analyses. *p* < 0.05 was considered to be statistically significant.

## Results

### Visual findings

**Fig. 1:**
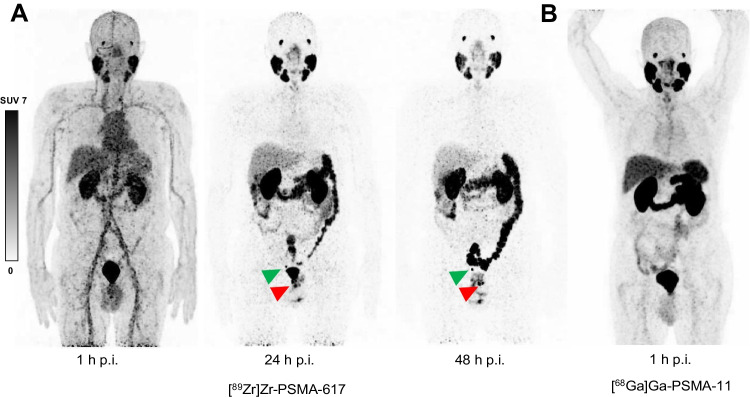
Maximum intensity projection (MIP) images of a patient with biochemical recurrence of prostate cancer (PSA 2.5 ng/mL, PSA doubling time > 12 months at time of imaging) on a) [^89^Zr]Zr-PSMA-617 PET/CT 1 h, 24 h, and 48 h post-injection (p.i.) and b) [^68^Ga]Ga-PSMA-11 PET/CT 1 h post-injection. [^89^Zr]Zr-PSMA-617 PET/CT revealed a suspected local recurrence (red arrow) and a suspected pelvic lymph node metastasis (green arrow) on 24-h and 48-h post-injection scans, findings that were not discerned on [^68^Ga]Ga-PSMA-11 PET/CT

Eighteen of the 23 patients (78%) included in this analysis had clear visual evidence of lesions suspicious for prostate cancer on one or more [^89^Zr]Zr-PSMA-617 PET/CT scans, while 5 (22%) had no such findings (Table 2). PSA ranged from 0.19 ng/mL to 2.5 ng/mL in the patients with positive scans and from 0.11 ng/mL to 1.55 ng/mL in the patients with negative scans. Figure 1 shows representative [^89^Zr]Zr-PSMA-617 PET images 1 h, 24 h, and 48 h post-injection from a patient with a positive scan.

**Table 2:** [^89^Zr]Zr-PSMA-617 PET/CT findings

Variable	Value		
Suspicious lesions on [^89^Zr]Zr-PSMA-617 PET/CT	24-h scan	48-h scan	
Number of patients with suspicious lesions	16	18	
Number of lesions			
Any	33	36	
Local recurrence	8	11	
Lymph node metastasis	21	21	
Bone metastasis	4	4	
[^89^Zr]Zr-PSMA-617 PET variables, mean ± SD [min.–max.]			
SUV_max_			
	24-h scan	48-h scan	*p*-value
All lesions	12.7 ± 11.4 [3.2–54.8]	14.0 ± 12.5 [2.5–60.5]^a^ 13.6 ± 12.3 [2.5–60.5]^b^	0.104^a^
Local recurrence	15.3 ± 14.1 [3.2–44.5]	16.5 ± 15.5 [3.1–49.5]^a^ 14.7 ± 14.0 [3.1–49.5]^b^	0.55^a^
Lymph node metastasis	10.4 ± 6.0 [3.5–29.7]	11.6 ± 6.8 [2.5–25.5]	0.36
Bone metastasis	19.4 ± 23.7 [5.9–54.8]	21.7 ± 26.8 [7.4–60.5]	0.13
SUV_peak_			
	24-h scan	48-h scan	*p*-value
All lesions	3.7 ± 3.2 [0.8–14.5]	3.4 ± 2.8 [0.6–12.7]^a^ 3.3 ± 2.7 [0.6–12.7]^b^	0.01^a^
Local recurrence	4.9 ± 4.6 [1.4–14.5]	4.3 ± 4.2 [0.6–12.7]^a^ 3.8 ± 3.7 [0.6–12.7]^b^	0.016^a^
Lymph node metastasis	2.9 ± 1.6 [0.8–7.8]	2.8 ± 1.7 [0.7–7.3]	0.30
Bone metastasis	5.7 ± 5.3 [1.7–12.8]	4.9 ± 4.4 [1.5–11.0]	0.38
TBR_max_			
	24-h scan	48-h scan	*p*-value
All lesions	29.7 ± 24.9 [7.0–107.5]	64.8 ± 59.7 [8.4–291.2]^a^ 64.2 ± 59.2 [8.4–291.2]^b^	< 0.001^a^
Local recurrence	36.4 ± 30.7 [7.0–94.7]	85.2 ± 94.1 [8.4–291.2]^a^ 77.7 ± 84.8 [8.4–291.2]^b^	< 0.001^a^
Lymph node metastasis	25.4 ± 17.1 [7.7–80.3]	54.0 ± 35.7 [13.2–141.7]	< 0.001
Bone metastasis	39.2 ± 45.7 [12.6–107.5]	89.4 ± 81.6 [24.7–201.7]	0.13
TBR_peak_			
	24-h scan	48-h scan	*p*-value
All lesions	8.8 ± 7.2 [1.8–30.9]	15.8 ± 14.5 [2.3–74.7]^a^ 15.6 ± 14.2 [2.3–74.7]^b^	< 0.001^a^
Local recurrence	11.6 ± 9.9 [3.3–30.9]	21.9 ± 24.4 [2.3–74.7]^a^ 19.5 ± 21.6 [2.3–74.7]^b^	< 0.001^a^
Lymph node metastasis	7.1 ± 4.8 [1.9–21.1]	12.9 ± 8.5 [3.7–36.5]	< 0.001
Bone metastasis	11.9 ± 10.5 [3.3–25.1]	19.0 ± 13.8 [5.0–36.7]	0.13

max., maximum; min., minimum; PET/CT, positron emission tomography/computed tomography; PSMA, prostate-specific membrane antigen; SD, standard deviation; SUV_max_, maximum standardized uptake value; SUV_peak_, peak standardized uptake value; TBR_max_, tumor-to-background ratio of SUV_max_: SUV_max_ of presumed tumor lesion/SUV_mean_ of healthy gluteal muscle; TBR_peak_, tumor-to-background ratio of SUV_peak_: SUV_peak_ of presumed tumor lesion/SUV_mean_ of healthy gluteal muscle

^a^ Calculation based on lesions seen in both 24-h and 48-h post-injection scans

^b^ Calculation based on all lesions seen in 48-h post-injection scan

**Fig. 2:**
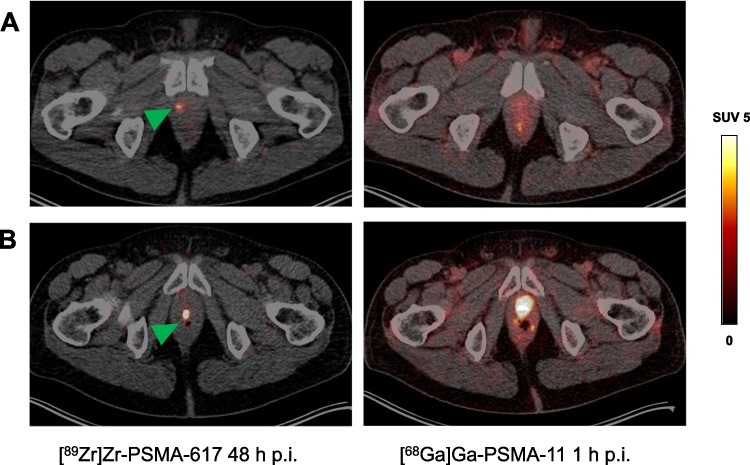
Transversal slice images showing presumed local recurrence of prostate cancer (green arrows) revealed by [^89^Zr]Zr-PSMA-617 PET/CT (left-hand column) but not identified on [^68^Ga]Ga-PSMA-11 PET/CT (right-hand column) in two patients (rows **A** and **B**, respectively). p.i., post-injection; SUV, standardized uptake value

**Fig. 3:**
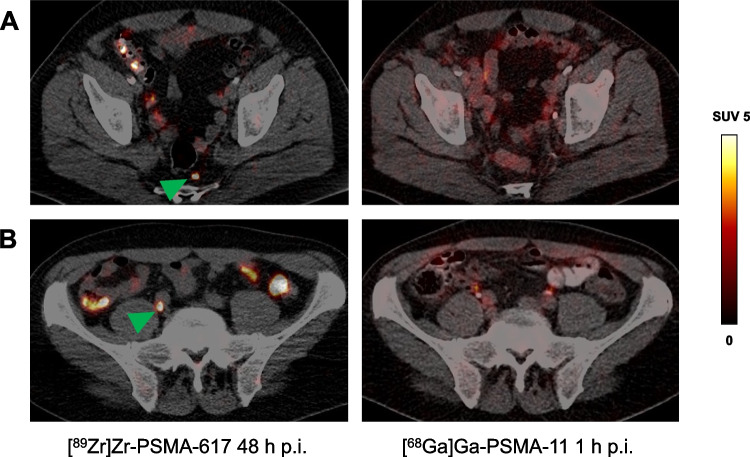
Transversal slice images showing presumed lymph node metastases of prostate cancer (green arrows) revealed by [^89^Zr]Zr-PSMA-617 PET/CT (left-hand column) but not identified on [^68^Ga]Ga-PSMA-11 PET/CT (right-hand column) in two patients (rows **A** and **B**, respectively). p.i., post-injection; SUV, standardized uptake value

**Fig. 4:**
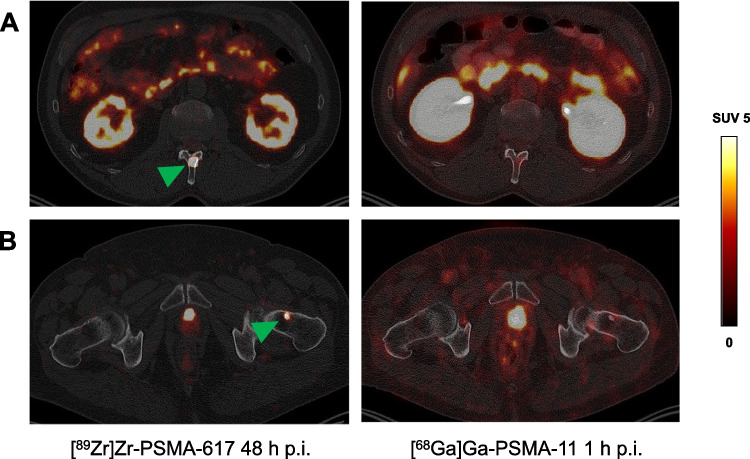
Transversal slice images showing suspected bone metastases of prostate cancer (green arrows) revealed by [^89^Zr]Zr-PSMA-617 PET/CT (left-hand column) but not discernible on [^68^Ga]Ga-PSMA-11 PET/CT (right-hand column) in two patients (rows **A** and **B**, respectively). p.i., post-injection; SUV, standardized uptake value

Altogether 36 lesions (minimum–maximum 1–4 per patient) were detected in the 18 patients with positive [^89^Zr]Zr-PSMA-617 PET/CT scans. Of these lesions, 11 were suspected to be local (prostate bed) recurrence, 21, lymph node metastases, and 4, bone metastases. Figures 2, 3 and 4 display representative images of lesions that were classified as either a local recurrence, a lymph node metastasis, or a bone metastasis.

Thirty-three lesions (92%) were visible on the 24-h scan, all of which also were visible on the 48-h scan. Three additional lesions (8%), all presumed to be local (prostate bed) recurrences, were seen only on the 48-h scan. Of these 36 lesions, only one, which was classified as a bone metastasis, was visible on the 1-h scan; this lesion was also noted on both the 24-h and 48-h scans.

### PET variables

**Fig. 5:**
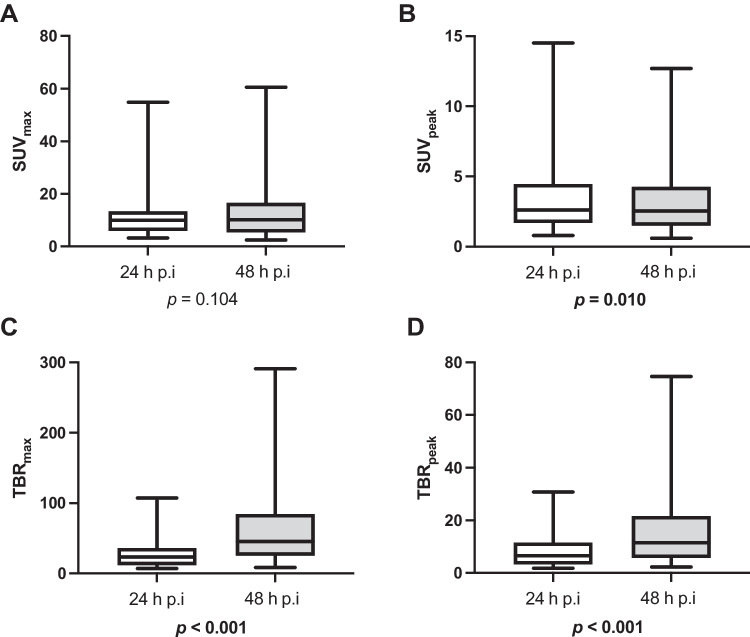
Aggregate descriptive statistics of PET variables of lesional radiotracer uptake and lesion-to-background ratio of all detected lesions suspicious for prostate cancer in [^89^Zr]Zr-PSMA-617 PET/CT images acquired at 24 h vs. 48 h post-injection: a) SUV_max_, b) SUV_peak_, c) TBR_max_, and d) TBR_peak_. *p* values refer to intra-lesion comparisons of the values between the 24-h and 48-h post-injection scans for all lesions visible on both scans (n = 33). SD, standard deviation; SUV_max_, maximum standardized uptake value; SUV_peak_, peak standardized uptake; TBR_max_, tumor-to-background ratio of SUV_max_: SUV_max_ of presumed tumor lesion/SUV_mean_ of healthy gluteal muscle; TBR_peak_, tumor-to-background ratio of SUV_peak_: SUV_peak_ of presumed tumor lesion/SUV_mean_ of healthy gluteal muscle

In intra-lesion comparisons involving all 33 lesions visible on both scans, SUV_max_ did not differ significantly between 24-h scans versus 48-h scans (*p* = 0.104), while SUV_peak_ was slightly but significantly lower in 48-h scans (*p* = 0.01) (Fig. 5). Results of PET variables across scans and lesion types are compiled in Table 2.

**Fig. 6:**
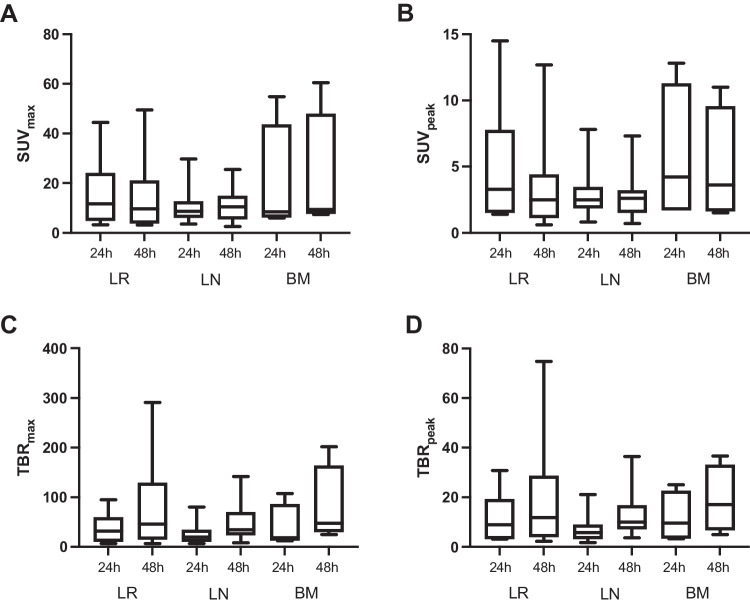
Aggregate statistics of PET variables of lesional radiotracer uptake and lesion–background ratio by lesion classification in [^89^Zr]Zr-PSMA-617 PET/CT images acquired 24 h post-injection and 48 h post-injection: a) SUV_max_, b) SUV_peak_, c) TBR_max_, and d) TBR_peak_. BM, bone metastases; LN, lymph node metastases; LR, local recurrence; SD, standard deviation; SUVmax, maximum standardized uptake value; SUVpeak, peak standardized uptake; TBR_max_, tumor-to-background ratio of SUV_max_: SUV_max_ of presumed tumor lesion/SUV_mean_ of healthy gluteal muscle; TBR_peak_, tumor-to-background ratio of SUV_peak_: SUV_peak_ of presumed tumor lesion/SUV_mean_ of healthy gluteal muscle

In contrast, intra-lesion comparisons, also involving all 33 lesions visible on both scans, of variables reflecting lesion-to-background ratio, i.e., TBR_max_ and TBR_peak_, showed that values were significantly higher in the 48-h scan versus the 24-h scan (both *p* < 0.001) (Table 2; Fig. 5). The pattern of improved lesion-to-background ratio from the 24-h scan to the 48-h scan also held true across lesion types (Table 2; Fig. 6).

### Safety

No adverse events, including clinically-relevant vital signs abnormalities, were noted during the [^89^Zr]Zr-PSMA-617 PET/CT or the 4 weeks thereafter.

### Follow-up

All patients with positive [^89^Zr]Zr-PSMA-617 PET/CT imaging (n = 18) received treatment due to scan findings. One patient underwent lymphadenectomy of a solitary nodal lesion. The surgical specimen was histopathologically confirmed to contain prostate cancer using immunohistochemistry. The other patients underwent radiotherapy with the dose and fields based on the [^89^Zr]Zr-PSMA-617 scan observations (n = 15) or received systemic antiandrogen therapy (n = 2). In the patients who received radiotherapy, PSA decreased following that intervention in all cases, by an average of 72% ± 25%. Post-radiotherapy, the PSA value became undetectable in 5 of the 15 patients, decreased by > 70% in an additional 5/15, and decreased by 30–70% in the remaining 5/15. Post-treatment PSA became also undetectable in the 2 men given antiandrogen therapy.

## Discussion

This analysis of experience with [^89^Zr]Zr-PSMA-617 PET/CT involves, to our knowledge, the largest yet published cohort of men with BCR and recent negative conventional PSMA-targeted imaging. The study had four principal findings. First, we confirmed in a 23-patient sample the efficacy of [^89^Zr]Zr-PSMA-617 PET/CT in localizing lesions suspicious for prostate cancer, with a patient-level detection rate of 78% (18/23). The lesions detected were suggestive of the three most common forms of recurrent prostate cancer, local recurrence, lymph node metastasis, and bone metastasis. As would be expected in the BCR setting, each patient had a limited number (1–4 per patient) of suspicious lesions, i.e., structural correlates of the PSA elevation appear to have been detected at the oligometastatic stage or earlier. Notably, high detection efficacy was shown in this study despite low PSA concentrations (median 0.54 ng/mL, minimum–maximum 0.11–2.5 ng/mL).

Second, our analysis of PET variables of lesional tracer uptake and lesion–to-background ratio confirmed a pair of earlier observations regarding [^89^Zr]Zr-PSMA-617 pharmacokinetics [19]. Namely, lesional accumulation of this tracer remains broadly stable in the 24-h to 48-h post-administration interval, reflected in the present analysis by the lack of significant differences in SUV_max_ and by only slightly (albeit significantly) lower SUV_peak_ at 48 h. On the other hand, decreased physiological accumulation of [^89^Zr]Zr-PSMA-617 during that period resulted in an increased lesion-to-background ratio in the later scans, in the form of a statistically-significant, and roughly twofold higher, TBR_max_ and TBR_peak_ at 48 h vs 24 h. These pharmacokinetics likely explain our detection of 33 lesions on both the 24-h and 48-h scans, but 3 additional lesions only on 48-h images. Indeed, these pharmacokinetic findings, along with the persistent visibility on 48-h scans of all lesions seen on 24-h scans, and the greater detection efficacy of the later imaging, suggest that 48-h imaging may be preferable to 24-h imaging. However, it remains for future studies to more definitively determine the most appropriate timing for [^89^Zr]Zr-PSMA-617 PET/CT scanning.

Third, we found evidence that patients with suspicious lesions on [^89^Zr]Zr-PSMA-617 PET/CT appeared to have had true-positive scans. One patient underwent surgery based on scan findings, and true positivity was confirmed by surgical specimen histology and by PSA decreases following this procedure, a lymphadenectomy of a solitary nodal lesion. Further, in all 15 additional patients receiving salvage radiotherapy based on [89Zr]Zr-PSMA-617 PET/CT findings, PSA decreased afterwards. The average PSA reduction was 72% ± 25% and the minimum decrease, 30%, with the analyte becoming undetectable in 5 of these men.

Fourth, our analysis supported previous published observations of the safety of [^89^Zr]Zr-PSMA-617 PET/CT, at least over the short term [19]. No undesirable effects were noted by health care professionals or patients up to 4 weeks post-scanning.

Our observations are quite comparable to those of Dietlein et al. using PET/CT with another ^89^Zr-based radiotracer, [^89^Zr]Zr-PSMA-Df [13]. These investigators visually identified altogether 15 suspicious lesions (1–4 per patient) in 8/14 men (57%) with BCR and median (minimum–maximum) PSA of 0.85 (0.31–7.2) ng/mL. The [^89^Zr]Zr-PSMA-Df scans were acquired 24–144 h post-injection, within 5 weeks after negative [^68^Ga]Ga-PSMA-11 PET/CT (n = 4) or [^18^F]-JK-PSMA-7 PET/CT (n = 10). Aligned with our experience, Dietlein and colleagues also detected putative local recurrence, lymph node metastasis, and/or distant metastasis. Additionally, like us, these investigators reported no side effects of the novel imaging procedure.

Limitations of the present work should be kept in mind. This was a single-center, retrospective analysis of a relatively small number of patients, which merits a caveat regarding generalizability. Additionally, in the everyday practice setting reported here, malignancy of 35/36 suspected prostate cancer lesions was not assessed histopathologically. However, 1 patient who underwent excision of a solitary nodal lesion seen on [^89^Zr]Zr-PSMA-617 PET/CT had this finding histologically confirmed as prostate cancer. Additionally, true scan positivity was suggested by marked to complete biochemical responses following salvage radiotherapy in all 15 additional patients who received that intervention. Nonetheless, presence of detectable PSA post-radiotherapy in some of these men means that the presence of lesions missed by [^89^Zr]Zr-PSMA-617 PET/CT as well as by [^68^Ga]Ga-PSMA-11 PET/CT cannot be excluded. Thus, to more definitively characterize the diagnostic performance of this novel radiotracer, it will be useful to more systematically analyze biochemistry, imaging, and clinical follow-up after [^89^Zr]Zr-PSMA-617 PET/CT.

Additionally, [^68^Ga]Ga-PSMA-11 PET acquisition at delayed time points, e.g., 3 h post-injection was not performed in our cohort. Further, it cannot be excluded that not only the different radionuclides used in this study, but also the different PSMA ligands played a role in our results. Compared with PSMA-11, PSMA-617 has a longer persistence in the blood pool and is associated with higher background in PET images. However, no clinically relevant differences in tumor uptake of these ligands have been reported, thus an impact on our results seems to be unlikely [24]. Lastly, we did not evaluate longer-term safety of this imaging procedure, or comprehensively assess short-term safety. However, some reassurance is provided by safety having been demonstrated using zirconium-labeled radiopharmaceuticals in other settings [25–28].

[^89^Zr]Zr-PSMA-617 PET/CT may ultimately be somewhat of a “niche modality”, largely reserved for patients with negative or indeterminate conventional (early) PET/CT despite BCR. In such cases, [^89^Zr]Zr-PSMA-617 may be particularly suitable for patients wishing to avoid systemic treatment in favor of metastasis-directed approaches [13, 29–32]. The potentially treatment-altering and outcome-altering information to be gained from the examination would seem to substantially outweigh its disadvantage of an approximately 2.5 times higher radiation exposure than that associated with conventional PSMA PET/CT [19], and to justify application of this novel procedure, especially in the above-mentioned cases. Given the identical PSMA ligands in this pair of radiopharmaceuticals, another potential application of [^89^Zr]Zr-PSMA-617 PET/CT may be for pre-lutetium-177-PSMA-617 RLT dosimetry, to attempt to individualize, and thereby optimize, dosing to improve safety and efficacy.

## Conclusions

In men with BCR, even when PSA levels are low, [^89^Zr]Zr-PSMA-617 PET/CT seems to be effective (positivity rate 78% in our cohort) in detecting lesions suspicious for local recurrence and metastasis that eluded detection using conventional imaging such as [^68^Ga]Ga-PSMA-11 PET/CT. Prospective study of this novel imaging modality in men with BCR is warranted.

## Data Availability

The datasets used and analyzed during the current study are available from the corresponding author on reasonable request.

## Abbreviations

^18^F: Fluorine-18
^68^Ga: Gallium-68
^89^Zr: Zirconium-89
ADT: Androgen deprivation therapy
CT: Computed tomography
DM: Distant metastases
LR: Local recurrence
LN: Lymph node metastases
max.: Maximum
min.: Minimum
MIP: Maximum intensity projection
PET: Positron emission tomography
p.i.: Post-injection
PSA: Prostate-specific antigen
PSMA: Prostate-specific membrane antigen
RLT: Radioligand therapy
SD: Standard deviation
SUV_max_: Maximum standardized uptake value
SUV_mean_: Mean standardized uptake value
SUV_peak_: Peak standardized uptake value
TBR_max_: Tumor-to-background ratio of SUV_max_: SUV_max_ of presumed tumor lesion/SUV_mean_ of healthy gluteal muscle
TBR_peak_: Tumor-to-background ratio of SUV_peak_: SUV_peak_ of presumed tumor lesion/SUV_mean_ of healthy gluteal muscle

## References

[1] Hofman MS, Lawrentschuk N, Francis RJ, Tang C, Vela I, Thomas P (2020). Prostate-specific membrane antigen PET-CT in patients with high-risk prostate cancer before curative-intent surgery or radiotherapy (proPSMA): a prospective, randomised, multicentre study. Lancet.

[2] Afshar-Oromieh A, da Cunha ML, Wagner J, Haberkorn U, Debus N, Weber W (2021). Performance of [(68)Ga]Ga-PSMA-11 PET/CT in patients with recurrent prostate cancer after prostatectomy-a multi-centre evaluation of 2533 patients. Eur J Nucl Med Mol Imaging.

[3] Wright GL, Haley C, Beckett ML, Schellhammer PF (1995). Expression of prostate-specific membrane antigen in normal, benign, and malignant prostate tissues. Urol Oncol.

[4] Silver DA, Pellicer I, Fair WR, Heston WD, Cordon-Cardo C (1997). Prostate-specific membrane antigen expression in normal and malignant human tissues. Clin Cancer Res.

[5] Sweat SD, Pacelli A, Murphy GP, Bostwick DG (1998). Prostate-specific membrane antigen expression is greatest in prostate adenocarcinoma and lymph node metastases. Urology.

[6] Perera M, Papa N, Roberts M, Williams M, Udovicich C, Vela I (2020). Gallium-68 Prostate-specific Membrane Antigen Positron Emission Tomography in Advanced Prostate Cancer-Updated Diagnostic Utility, Sensitivity, Specificity, and Distribution of Prostate-specific Membrane Antigen-avid Lesions: A Systematic Review and Meta-analysis. Eur Urol.

[7] Pienta KJ, Gorin MA, Rowe SP, Carroll PR, Pouliot F, Probst S, et al. A Phase 2/3 prospective multicenter study of the diagnostic accuracy of prostate specific membrane antigen PET/CT with (18)F-DCFPyL in prostate cancer patients (OSPREY). J Urol. 2021;206:52–61. 10.1097/JU.0000000000001698. 10.1097/JU.0000000000001698 PMC8556578 33634707

[8] Morris MJ, Rowe SP, Gorin MA, Saperstein L, Pouliot F, Josephson D, et al. diagnostic performance of (18)F-DCFPyL-PET/CT in men with biochemically recurrent prostate cancer: Results from the CONDOR Phase III, multicenter study. Clin Cancer Res. 2021. 10.1158/1078-0432.CCR-20-4573. 10.1158/1078-0432.CCR-20-4573 PMC8382991 33622706

[9] Calais J, Czernin J, Fendler WP, Elashoff D, Nickols NG (2019). Randomized prospective phase III trial of (68)Ga-PSMA-11 PET/CT molecular imaging for prostate cancer salvage radiotherapy planning [PSMA-SRT]. BMC Cancer.

[10] Giesel FL, Hadaschik B, Cardinale J, Radtke J, Vinsensia M, Lehnert W (2017). F-18 labelled PSMA-1007: biodistribution, radiation dosimetry and histopathological validation of tumor lesions in prostate cancer patients. Eur J Nucl Med Mol Imaging.

[11] Piron S, Verhoeven J, Vanhove C, De Vos F (2022). Recent advancements in (18)F-labeled PSMA targeting PET radiopharmaceuticals. Nucl Med Biol.

[12] Wurzer A, DiCarlo D, Schmidt A, Beck R, Eiber M, Schwaiger M (2019). Radiohybrid ligands: a novel tracer concept exemplified by (18)F- or (68)Ga-labeled rhPSMA-inhibitors. J Nucl Med.

[13] Dietlein F, Kobe C, Vazquez SM, Fischer T, Endepols H, Hohberg M, et al. An (89)Zr-labeled PSMA tracer for PET/CT imaging of prostate cancer patients. J Nucl Med. 2022;63:573–83. 10.2967/jnumed.121.262290. 10.2967/jnumed.121.262290 34326129

[14] Giesel FL, Knorr K, Spohn F, Will L, Maurer T, Flechsig P, et al. Detection efficacy of (18)F-PSMA-1007 PET/CT in 251 patients with biochemical recurrence of prostate cancer after radical prostatectomy. J Nucl Med. 2019;60:362–8. 10.2967/jnumed.118.212233. 10.2967/jnumed.118.212233 PMC6424235 30042163

[15] Burgard C, Hoffmann MA, Frei M, Buchholz HG, Khreish F, Marlowe RJ, et al. Detection efficacy of (68)Ga-PSMA-11 PET/CT in biochemical recurrence of prostate cancer with very low PSA levels: a 7-year, two-center “real-world” experience. Cancers (Basel). 2023;15:1376. 10.3390/cancers15051376. 10.3390/cancers15051376 PMC10000220 36900169

[16] Afshar-Oromieh A, Sattler LP, Mier W, Hadaschik BA, Debus J, Holland-Letz T, et al. The clinical impact of additional late PET/CT imaging with (68)Ga-PSMA-11 (HBED-CC) in the diagnosis of prostate cancer. J Nucl Med. 2017;58:750–5. 10.2967/jnumed.116.183483. 10.2967/jnumed.116.183483 28062595

[17] Prive BM, Derks YHW, Rosar F, Franssen GM, Peters SMB, Khreish F (2022). (89)Zr-labeled PSMA ligands for pharmacokinetic PET imaging and dosimetry of PSMA-617 and PSMA-I&T: a preclinical evaluation and first in man. Eur J Nucl Med Mol Imaging.

[18] Vazquez SM, Endepols H, Fischer T, Tawadros SG, Hohberg M, Zimmermanns B, et al. Translational development of a Zr-89-labeled inhibitor of prostate-specific membrane antigen for PET imaging in prostate cancer. Mol Imaging Biol. 2022;24:115–25. 10.1007/s11307-021-01632-x. 10.1007/s11307-021-01632-x PMC8760230 34370181

[19] Rosar F, Schaefer-Schuler A, Bartholoma M, Maus S, Petto S, Burgard C (2022). [(89)Zr]Zr-PSMA-617 PET/CT in biochemical recurrence of prostate cancer: first clinical experience from a pilot study including biodistribution and dose estimates. Eur J Nucl Med Mol Imaging.

[20] Pandya DN, Bhatt N, Yuan H, Day CS, Ehrmann BM, Wright M (2017). Zirconium tetraazamacrocycle complexes display extraordinary stability and provide a new strategy for zirconium-89-based radiopharmaceutical development. Chem Sci.

[21] Pandya DN, Bhatt NB, Almaguel F, Rideout-Danner S, Gage HD, Solingapuram Sai KK, et al. (89)Zr-chloride can be used for immuno-PET radiochemistry without loss of antigen reactivity in vivo. J Nucl Med. 2019;60:696–701. 10.2967/jnumed.118.216457. 10.2967/jnumed.118.216457 PMC6495241 30442753

[22] Rosar F, Bartholoma M, Maus S, Prive BM, Khreish F, Franssen GM, et al. 89Zr-PSMA-617 PET/CT may reveal local recurrence of prostate cancer unidentified by 68Ga-PSMA-11 PET/CT. Clin Nucl Med. 2022;47:435–6. 10.1097/RLU.0000000000004108. 10.1097/RLU.0000000000004108 35234197

[23] Fendler WP, Eiber M, Beheshti M, Bomanji J, Ceci F, Cho S (2017). (68)Ga-PSMA PET/CT: Joint EANM and SNMMI procedure guideline for prostate cancer imaging: version 10. Eur J Nucl Med Mol Imaging.

[24] Guhne F, Radke S, Winkens T, Kuhnel C, Greiser J, Seifert P, et al. Differences in distribution and detection rate of the [(68)Ga]Ga-PSMA ligands PSMA-617, -I&T and -11-inter-individual comparison in patients with biochemical relapse of prostate cancer. Pharmaceuticals (Basel). 2021;15:9. 10.3390/ph15010009. 10.3390/ph15010009 PMC8779232 35056066

[25] Yoon JK, Park BN, Ryu EK, An YS, Lee SJ. Current perspectives on (89)Zr-PET imaging. Int J Mol Sci. 2020;21:4309 10.3390/ijms21124309. 10.3390/ijms21124309 PMC7352467 32560337

[26] Pandit-Taskar N, O'Donoghue JA, Beylergil V, Lyashchenko S, Ruan S, Solomon SB (2014). (8)(9)Zr-huJ591 immuno-PET imaging in patients with advanced metastatic prostate cancer. Eur J Nucl Med Mol Imaging.

[27] Pandit-Taskar N, O’Donoghue JA, Durack JC, Lyashchenko SK, Cheal SM, Beylergil V, et al. A phase I/II study for analytic validation of 89Zr-J591 immunoPET as a molecular imaging agent for metastatic prostate cancer. Clin Cancer Res. 2015;21:5277–85. 10.1158/1078-0432.CCR-15-0552. 10.1158/1078-0432.CCR-15-0552 PMC4668231 26175541

[28] Pandit-Taskar N, Postow MA, Hellmann MD, Harding JJ, Barker CA, O’Donoghue JA, et al. First-in-humans imaging with (89)Zr-Df-IAB22M2C anti-CD8 minibody in patients with solid malignancies: preliminary pharmacokinetics, biodistribution, and lesion targeting. J Nucl Med. 2020;61:512–9. 10.2967/jnumed.119.229781. 10.2967/jnumed.119.229781 PMC7198374 31586002

[29] Deek MP, Van der Eecken K, Sutera P, Deek RA, Fonteyne V, Mendes AA, et al. Long-term outcomes and genetic predictors of response to metastasis-directed therapy versus observation in oligometastatic prostate cancer: analysis of STOMP and ORIOLE trials. J Clin Oncol. 2022;40:3377–82. 10.1200/JCO.22.00644. 10.1200/JCO.22.00644 PMC10166371 36001857

[30] Jadvar H, Abreu AL, Ballas LK, Quinn DI. Oligometastatic prostate cancer: current status and future challenges. J Nucl Med. 2022;63:1628–35. 10.2967/jnumed.121.263124. 10.2967/jnumed.121.263124 PMC9635685 36319116

[31] von Deimling M, Rajwa P, Tilki D, Heidenreich A, Pallauf M, Bianchi A (2022). The current role of precision surgery in oligometastatic prostate cancer. ESMO Open.

[32] Berghen C, Joniau S, Ost P, Poels K, Everaerts W, Decaestecker K, et al. Progression-directed therapy for oligoprogression in castration-refractory prostate cancer. Eur Urol Oncol. 2021;4:305–9. 10.1016/j.euo.2019.08.012. 10.1016/j.euo.2019.08.012 31558422

